# Improved Cell-Free RNA and Protein Synthesis System

**DOI:** 10.1371/journal.pone.0106232

**Published:** 2014-09-02

**Authors:** Jun Li, Liangcai Gu, John Aach, George M. Church

**Affiliations:** Department of Genetics, Harvard Medical School, Boston, Massachusetts, United States of America; Universität Stuttgart, Germany

## Abstract

Cell-free RNA and protein synthesis (CFPS) is becoming increasingly used for protein production as yields increase and costs decrease. Advances in reconstituted CFPS systems such as the Protein synthesis Using Recombinant Elements (PURE) system offer new opportunities to tailor the reactions for specialized applications including *in vitro* protein evolution, protein microarrays, isotopic labeling, and incorporating unnatural amino acids. In this study, using firefly luciferase synthesis as a reporter system, we improved PURE system productivity up to 5 fold by adding or adjusting a variety of factors that affect transcription and translation, including Elongation factors (EF-Ts, EF-Tu, EF-G, and EF4), ribosome recycling factor (RRF), release factors (RF1, RF2, RF3), chaperones (GroEL/ES), BSA and tRNAs. The work provides a more efficient defined *in vitro* transcription and translation system and a deeper understanding of the factors that limit the whole system efficiency.

## Introduction

As an alternative to *in vivo* protein synthesis, CFPS systems provide the ability to produce a variety of compounds and present numerous advantages: concentrations of some system components can be controlled, therefore a large parameter space can be studied [1], [2]; toxic products to a host cell, such as membrane proteins, can be produced with better yields [3]. Due to the high engineering flexibility of CFPS, they are applied to high-throughput methodologies [4]–[6], non-standard amino acids incorporation into proteins, and *in vitro* protein evolution [7].

Cell-free translation has been achieved by two approaches. One approach is based on crude cell extract, derived from *Escherichia coli*, wheat germ or rabbit reticulocytes [8]–[12]. Cell-free systems based on crude extracts have been optimized for long-lived synthesis and high yield [13], [14], but their range of applications is still limited by their complicated nature. Issues include independent of peptide bond formation [15], and template nucleic acids or protein products degradation by nucleases or proteases [16]. The other approach is reconstituting well-defined protein synthesis systems from recombinant factors. The “Protein synthesis Using Recombinant Elements” (PURE) system is a partially recombinant, CFPS system reconstituted solely from elements essential to *E. coli* translation [17]. The PURE system does not contain some of the detrimental enzymes found in extracts. It provides higher reaction controllability in comparison to crude extract-based CFPS systems for translation studies and biotechnology applications [18]. Table 1 systematically compares the PURE system with a commercialized *E. coli* crude extract CFPS (the 5 PRIME RTS system). The ribosome concentration is ∼2.4 µM in the PURE system and ∼1.6 µM in the crude extract system, The PURE system's productivity as measured by the yield of active firefly luciferase is 3 fold lower [19] while its cost is ∼4 times higher per gram of protein produced than those of the crude extract system. Additionally, the PURE system's preparation procedure, which involves multiple column based purifications, is more labor and time consuming than the preparation of crude extract system. To simplify the preparation procedure, our group applied the Multiplex Automated Genome Engineering (MAGE) method to insert hexa-histidine sequences into 38 essential genes *in vivo* that encode the entire translation machinery of *E. coli*, and produced a streamlined, co-purified, and reconstituted PURE system *in vitro* that is about 11% as active as a commercial system (New England Biolabs) [20]. Therefore developing a cost-effective PURE system with higher productivity is necessary.

**Table 1:** Comparison of PURE system with RTS system (*E. coli* crude cell extract based cell-free system).

	5 PRIME RTS System (*E. coli* crude cell extract)	PURE System
**Productivity**	2–20 µg/50 µL reaction	0.5–10 µg/50 µL reaction
**System composition**	Undefined, proteases and nucleases and tmRNA exist	Defined
**Ribosome concentration**	∼1.6 µM	∼2.4 µM
**Engineering flexibility**	low	high
**Linear template tolerance**	low	high
**Preparation**	Centrifuge based, quick and simple	Multiple column based purifications, labor intensive and time consuming
**Price**	$1.2–12/µg protein	$4.4–88/µg protein
**Translation efficiency**	higher	lower

System productivity and ribosome concentration information were all obtained from 5 PRIME RTS system and PURE system handbook.

Ignoring energy supply and small molecule metabolism, the most common focus of CFPS enhancement efforts, several factors have been identified that improve *E. coli* crude extract CFPS yield and stability. It has been shown [21] that adding purified elongation factors (EFs) to an *E. coli* crude extract CFPS (a modified PANOx-SP system [22]) increases protein synthesis rates and yields by increasing both translation initiation and elongation rates. Multiple groups have identified improvements by studying translation dynamics in a simplified PURE system that lacks transcription and tRNA synthesis [23]–[25]. For instance, Pavlov, M. Y etc. reported that ribosome recycling times are minimized when RF1 concentrations are slightly smaller than the total ribosome concentration in an *in vitro* translation system [23]. Chaperone systems (e.g., GroEL/ES and DnaK/DnaJ/GrpE) were shown to alleviate protein aggregation when added to the PURE system [26]–[28], but their effects on the yield of different functional proteins are yet to be characterized. Recently identified Elongation Factor 4 (EF4), which induces back-translocations in ribosomes that have experienced defective translocations, was also shown to affect *E. coli* crude extract CFPS productivity [29]. Compared to cytosol, which has a protein concentration of 200–300 g/L and a RNA concentration of 75–150 g/L, cell-free systems are very dilute. Crowding agents PEG-8000, Ficoll-70 and Ficoll-400, when added to cell-free systems at certain concentrations, have been found to boost transcription but inhibit translation [30].

Notably, the non-energy system factors described above have been explored mainly one at a time in a variety of CFPS systems, and little effort has been spent trying to consolidate these improvements within a single system. Here, using firefly luciferase (Fluc) as a reporter, we optimized the commercialized PURE system productivity by adjusting several factors individually and in combination. We report that we were able to boost the yield of functional Fluc between 25% and 70% through increases of EF-Ts, EF-Tu and EF-G concentrations; adjustments to ribosome recycling factor (RRF) and release factor [31] 1, 2, 3 concentrations; addition of EF4, GroEL/ES, and BSA; and increasing tRNA concentration; these factors taken individually or in small subsets. By combining all of our individual optimizations, we achieved a >5 fold increase in PURE system productivity as measured by the yield of functional Fluc. We further investigated these factors on the synthesis of a fluorescence protein mCherry and an *E. coli* protein β-galactosidase (β-gal) in the PURE system and achieved ∼3.2 and ∼2.5 fold active product yield, respectively.

## Results

### Improving PURE system translation by adding translation factors

The PURE system contains IF1, IF2, IF3, EF-Tu, EF-Ts, EF-G, RF1, RF3, RRF, 20 aminoacyl-tRNA synthetases (ARSs), methionyl-tRNA transformylase (MTF), T7 RNA polymerase, ribosomes, 46 tRNAs, NTPs, creatine phosphate, 10-formyl-5,6,7,8-tetrahydrofolic acid, 20 amino acids, creatine kinase, myokinase, nucleoside-diphosphate kinase, and pyrophosphatase [17], [32]. Transcription and translation in PURE system are two consecutive processes. We investigated the overall outcome of the two processes as measured by functional Fluc produced under a variety of conditions using a pIVEX 2.3d-Fluc plasmid harboring Fluc gene under T7 promoter and terminator control. Reactions were conducted for 2 hours at 37°C. The amount of functional Fluc produced was measured in relative luminescence units by a microplate reader.

**Figure 1:**
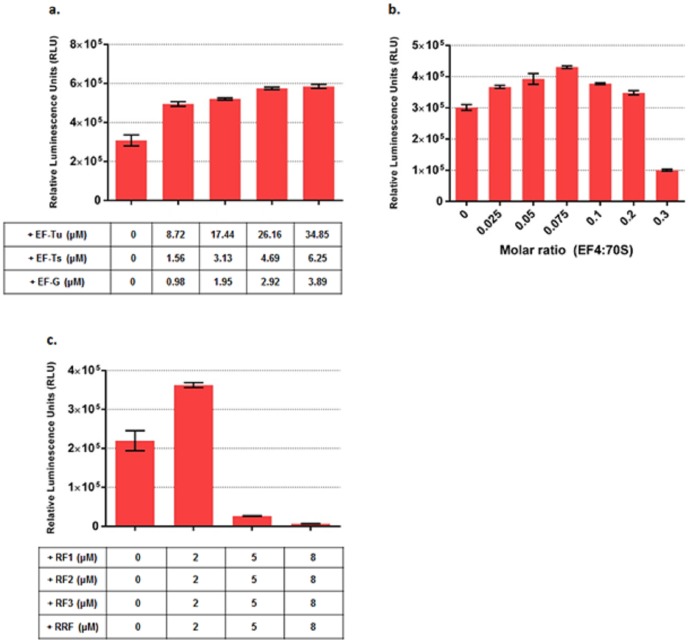
Optimization of PURE system as measured by active Fluc produced by supplementing different concentrations of EF-Tu, Ts, G; EF4; RF1, 2, 3 and RRF. **(a).** Active Fluc produced at different EF-Ts, Tu and G concentrations. The table below shows the actual concentration increase of EF-Ts, Tu and G in the PURE system. **(b).** Active Fluc produced at different EF4 concentrations. **(c).** Active Fluc produced at different RF1, 2, 3 and RRF concentrations. The table below shows the actual concentration increase of RF1, 2, 3 and RRF in the PURE system. Fluc activities were measured in relative luminescence unit by luciferase assay and PURE system reaction without supplement was set as control. Error bars are ± standard deviations, with n = 3.

We first increased the concentration of EFs which are already present in the system and found translation was enhanced by the addition of EF-Tu, Ts and G (Figure 1a). Over a series of stepwise, proportional additions of EF-Tu, Ts and G, we observed a maximum increase of ∼60% functional Fluc when concentrations of EF-Tu, Ts and G were simultaneously increased by 34.85 µM, 6.25 µM and 3.89 µM respectively.

The newly identified EF4 gene lepA was cloned from *E. coli* strain MG1655 to a pET-24b vector with a C-terminal hexa-histidine (his) tag and overexpressed in BL21 (DE3) *E. coli* cells. EF4 was then purified with ÄKTAprime (GE Healthcare) equipped with 5 mL HisTrap HP column and analyzed on a 4–12% Bis-Tris PAGE gel (Figure S1). The electrophoretic pattern indicated that EF4 was purified to homogeneity. As increased amounts of EF4 were added to PURE system, the total functional Fluc amount increased and peaked at a ratio of 0.075 molecules EF4 added per 70 S. At the peak, EF4 improved PURE translation by ∼30% in terms of the yield of functional Fluc (Figure 1b). Further addition of EF4 led to a rapid reduction in the functional Fluc production, in agreement with the effects of EF4 in *E. coli* crude extract based CFPS [29].

We also increased RRF, RF1, 2, 3 concentrations by 2 µM, 5 µM and 8 µM. Compared to the original commercialized PURE system, we observed a maximum boost of ∼55% in functional Fluc yield when the concentrations were increased by 2 µM (Figure 1c). Further increase of RFs and RRF concentrations significantly decreased yield (Figure 1c).

To test whether adding translation factors has similar effects on the synthesis of other proteins, we constructed another two reporter proteins mCherry and β-gal into a pET-24b vector under T7 control and tested their expression in PURE system at different concentrations of translation factors. A maximum increase of ∼60% in active mCherry yield was observed when concentrations of EF-Tu, Ts and G were simultaneously increased by 17.44 µM, 3.13 µM and 1.95 µM respectively (Figure S2a). For β-gal, a maximum increase of ∼28% in active product yield was reached when EF-Tu, Ts and G concentrations were simultaneously increased by 8.72 µM, 1.56 µM, and 0.98 µM respectively (Figure S3a). At the optimized EF-Tu, Ts and G concentrations for Fluc synthesis, mCherry showed a boost of ∼30% in active product yield while β-gal showed a similar level of active product yield compared to the original PURE system (Figure S2a and S3a). When EF4 was added to the system the total active product yield for mCherry and β-gal increased and also peaked at a ratio of 0.075 molecules EF4 added per 70S. At the peak, it exhibited a ∼2 fold active product yield for mCherry and a ∼1.4 fold active product yield for β-gal compared to the original PURE system (Figure S2b and S3b). We also tried increasing the concentrations of RF1, RF2, RF3 and RRF when expressing mCherry and β-gal in the PURE system. The yield of active mCherry was maximally increased by 60% when RF1, RF2, RF3 and RRF concentrations were increased by 2 µM (Figure S2c). The yield of active β-gal was boosted maximally by ∼40% when RF1, RF2, RF3 and RRF concentrations were increased by 1 µM (Figure S3c).

### PURE system transcription and translation under macromolecular crowding conditions

**Figure 2:**
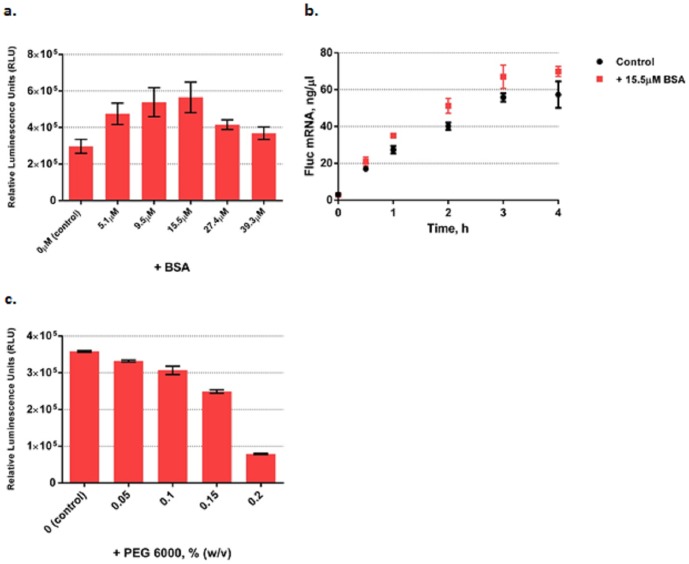
Optimization of PURE system as measured by functional Fluc produced by adding macromolecular crowding agents. **(a).** Active Fluc produced in the system at different BSA concentrations. **(b).** The time course (kinetics) of transcription measured by Fluc mRNA yields with the Quant-iT RiboGreen RNA reagent with and without the presence of 15.5 µM BSA. **(c).** Active Fluc produced in the system at different PEG-6000 concentrations. In (a) and (c) Fluc activities were measured in relative luminescence units by luciferase assay and PURE system reaction without supplement was set as control. Error bars are ± standard deviations, with n = 3.

The PURE system comprises a relatively dilute solution that does not benefit from the macromolecular crowding effects present in living cells. We therefore added BSA to increase the overall PURE system protein concentration. Adding BSA steadily increased functional Fluc production and reached a maximum boost of ∼70% at 15.5 µM (Figure 2a). Further addition of BSA led to a steady reduction in the functional Flucyield. Then we decoupled PURE system transcription and translation by performing the PURE reaction without ribosomes and tRNAs to study BSA's crowding effect on transcription only. Figure 2b shows the Fluc mRNA yields measured with the Quant-iT RiboGreen RNA reagent. The time course (kinetics) of transcription shows that in the presence of 15.5 µM BSA, transcription proceeded faster than in the original PURE system with a 20% increase in the initial transcription velocity (Figure 2b). Adding another macromolecular crowding agent PEG-6000 decreased functional Fluc yield (Figure 2c) possibly because macromolecular crowding facilitated protein aggregation rather than correct folding of nascent proteins [30], [33].

We also tested BSA's macromolecular crowding effects on mCherry and β-gal synthesis in the PURE system and observed a maximum active product yield of ∼2 fold for mCherry at 15.5 µM BSA and ∼1.2 fold for β-gal at 5.1 µM BSA (Figure S4).

### Improving PURE system translation by adding chaperone systems

Chaperone systems such as DnaK/DnaJ/GrpE and GroEL/GroES are understood to contribute to the folding of newly synthesized polypeptides, either interacting with these at the ribosome or shortly after their release [26], [28], [34]. Niwa *etc.* comprehensively evaluated the effects of GroEL/GroES and DnaK/DnaJ/GrpE on ∼800 aggregation-prone cytosolic *E. coli* proteins using PURE system and found DnaK and GroEL systems drastically increased the solubility of hundreds of proteins [35]. A reasonable inference is that adding such chaperones to the basic PURE system should not only enhance protein folding and solubility but increase functional product yield, and that this should obtain for exogenous as well as native *E. coli* proteins. A previous study showed that DnaK system enhanced the binding activity of a single-chain antibody synthesized in the PURE system while the GroEL/ES system showed little effect on that [36].

**Figure 3:**
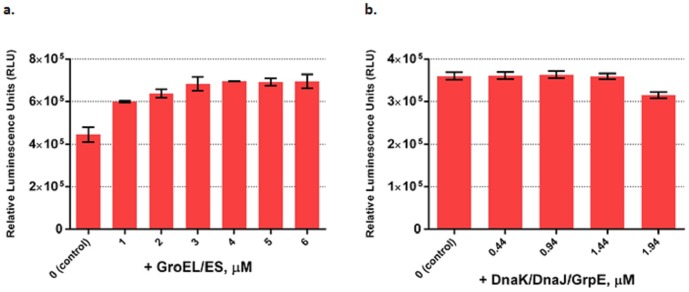
Optimization of PURE system as measured by functional Fluc produced by adding chaperone systems GroEL/ES and DnaK/DnaJ/GrpE. **(a).** Active Fluc produced at different GroEL/GroES concentrations. **(b).** Active Fluc produced at different DnaK/DnaJ/GrpE concentrations. Fluc activities were measured in relative luminescence unit by luciferase assay and PURE system reaction without supplement was set as control. Error bars are ± standard deviations, with n = 3.

Here we investigated the effect of adding these systems to PURE system on functional Fluc yield. Functional Fluc yield increased steadily as more GroEL/ES was added and reached a maximum increase of ∼60% with 4 µM GroEL/ES (Figure 3a). GroEL/ES also boosted active β-gal yield by ∼20% at a concentration of 4 µM (Figure S7a). However, it showed little positive effect on active product yield when applied to mCherry synthesis (Figure S6a). We also assessed total protein amount of Fluc produced at each concentration of GroEL/ES via SDS-PAGE (Figure S5). Since Fluc does not overlap in a Coomassie blue stained gel with any other protein present in the PURE system, the Fluc band can be scanned and an accurate determination of the total amount can be made. Interestingly, adding GroEL/ES also boosts the total amount of protein produced. When DnaK/DnaJ/GrpE system was added, it showed little positive effect on the yield of functional Fluc, mCherry and β-gal (Figure 3b, Figure S6b and S7b).

### Improving PURE system transcription and translation by adjusting tRNA, Mg^2+^ and ATP/GTP concentrations

We then studied the effects of adjusting tRNA, Mg^2+^ and ATP/GTP concentrations on PURE system protein synthesis with Fluc as the reporter. Here we incorporated adjustments to Mg^2+^ and ATP/GTP into versions of the PURE system that already included previously determined optimizations to EF-Ts, Tu, G, EF4, RF 1, 2, 3, RRF, GroEL/GroES and BSA for Fluc.

**Figure 4:**
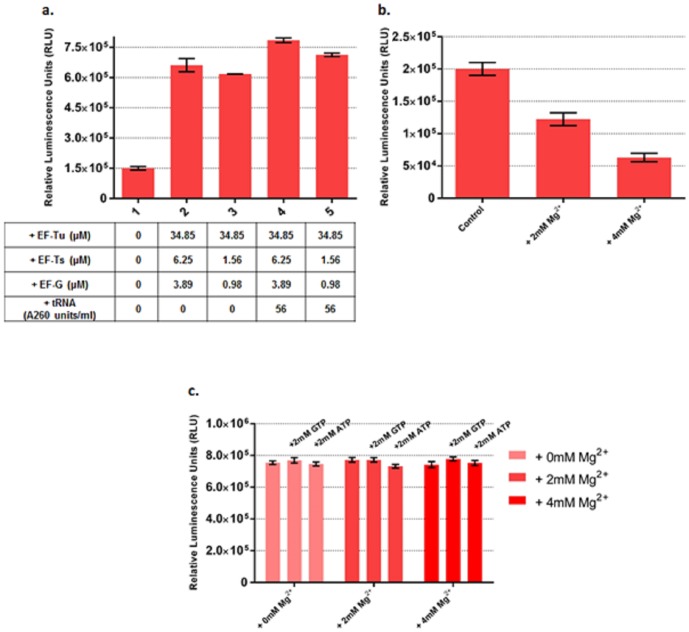
Optimization of PURE system as measured by functional Fluc produced by adjusting tRNA, ATP and GTP concentrations. **(a).** Increasing tRNA concentration by 56 A260 units/ml boosts functional Fluc yield by 25%. The table below shows the actual concentration increase of EF-Ts, Tu, G and tRNA in each reaction. Reaction 1 is taken as control. In reaction 2, 3, 4 and 5, EF4; RF 1, 2, 3, RRF; GroEL/GroES and BSA were added at their optimized concentrations. **(b).** Increasing Mg^2+^ concentration decreases functional Fluc yield in the original PURE system. **(c).** Increasing Mg^2+^, ATP and GTP concentrations has little effect on final yield of functional Fluc in our optimized PURE system with optimized concentrations of EF-Ts, Tu, G; EF4; RF 1, 2, 3, RRF; GroEL/GroES and BSA. Fluc activities were measured in relative luminescence unit by luciferase assay and PURE system reaction without supplement was set as control. Error bars are ± standard deviations, with n = 3.

When we followed this same procedure for testing adjusted tRNA concentrations, however, we included an additional combination of EF-Ts and Tu concentrations as well, where these were lower than the previously optimized values, holding the optimized concentrations of all other factors constant. Lowering EF-Ts and Tu concentrations in this way decreased the yield of the optimized system. Increasing tRNA concentration by 56 A260 units/mL in the system gave a 25% increase in functional Fluc yield for both concentration sets of EF-Ts, Tu (Figure 4a). Surprisingly, after combining the optimized concentrations of protein factors, increasing tRNA concentration by 56 A260 units/mL decreased the active product yield of mCherry and β-gal (Figure S8).

We first tested the effect of Mg^2+^ concentration on the original, unoptimized, PURE system. Adding more Mg^2+^ drastically decreased PURE system productivity as measured by active Fluc produced (Figure 4b). However, when we increased Mg^2+^ concentration by 2 mM and 4 mM and also increased ATP or GTP concentration by 2 mM at each concentration of Mg^2+^ in our optimized PURE system, we did not observe any significant changes in the yield of functional Fluc (Figure 4c). This suggests that our optimized PURE system exhibits reduced sensitivity to Mg^2+^ compared to the original PURE system.

### Assessment of improved PURE system by combining the optimized conditions

**Figure 5:**
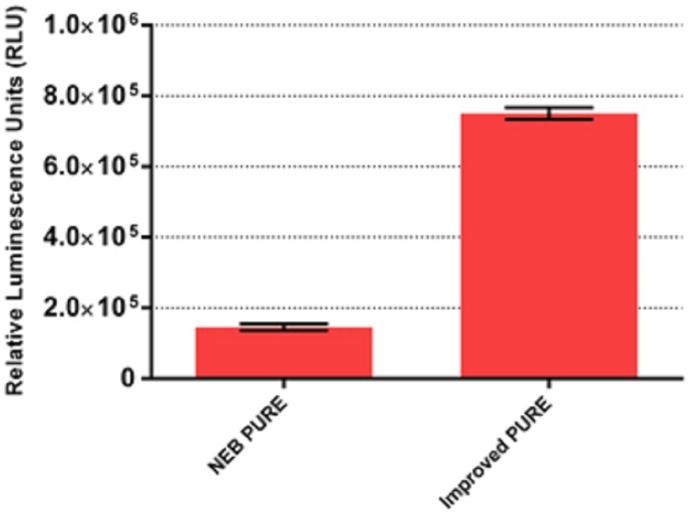
Optimization of PURE system with the best combination of EF-Ts, Tu, G; EF 4; RF 1,2,3, RRF; GroEL/GroES; BSA and tRNA concentrations as measured by functional Fluc produced. Fluc activities were measured in relative luminescence unit by luciferase assay and PURE system reaction without supplement was set as control. Error bars are ± standard deviations, with n = 3.

In the previous sections, we have optimized PURE system by individually assaying the protein factors and tRNA concentrations. Here we combined the individually optimized concentrations of EFs, RFs, RRF, GroEL/ES, BSA and tRNA and eventually achieved >5 fold increase in PURE system productivity as measured by the yield of functional Fluc compared to the original PURE system (Figure 5). By combining the individually optimized concentrations of EFs, RRF, RFs, and BSA for mCherry, we achieved a ∼3.2 fold active product yield (Figure S8a). When the individually optimized concentrations of EFs, RRF, RFs, GroEL/ES and BSA for β-gal were combined, a ∼2.5 fold active product yield was achieved (Figure S8b).

## Discussion

In this study, we added or adjusted the concentration of a variety of factors that affect transcription and translation and achieved a boost in PURE system productivity of more than 5 fold as measured by functional Fluc produced. The work provides a deeper understanding of cell-free system efficiency limitation factors. For both *E. coli* crude extract based cell-free system [21] and reconstituted PURE system, adding more elongation factors can boost the system productivity (Figure 1a) which indicates that system efficiency is limited by translation elongation capacity. In fact under rapid growth conditions, EF-Tu is the most abundant protein not only in *E. coli* but in most bacterial cells and reaches ∼10 times of the ribosome concentration to accelerate protein synthesis required by fast growth [37], [38]. The PURE system contains ∼2.4 µM ribosome. Adding EF-Tu to the system makes the EF-Tu to ribosome ratio more close to the ratio *in vivo* during rapid growth phase so that higher protein synthesis efficiency can be achieved. Moreover the concentrations of RF1, 2, 3 and RRF in the original PURE system are ∼0.25 µM, ∼0.24 µM, ∼0.17 µM and ∼0.485 µM, respectively. When we increased RF1, 2, 3 and RRF concentrations by 2 µM, the final concentrations of RF1, 2, 3 and RRF concentrations were close to the ribosome concentration 2.4 µM in PURE system. These steps presumably accelerated ribosome recycling by minimizing ribosome recycling time [23], [24] and resulted in a 55% increase in active Fluc yield (Figure 1c), suggesting that ribosome release and recycling are also limiting factors for PURE translation. It has also been reported that after the termination step of translation, the post-termination complex, composed of the ribosome, mRNA, and a deacylated tRNA, is processed by the concerted action of RRF, EF-G, GTP and probably IF3 to prepare the ribosome for a fresh round of protein synthesis [39]. We tested this effect on Fluc synthesis by increasing the concentrations of these three factors by 2 µM and eventually achieved a ∼30% boost in active Fluc yield (Figure S9a). Surprisingly, when we only increased IF1, 2, 3 concentrations by 2 µM and 4 µM, Fluc translation was severely inhibited (Figure S9b).

Another interesting finding is that supplementing chaperone system GroEL/ES not only improved the yield of functional Fluc (Figure 3a) but also increased the total amount of Fluc expressed (Figure S5). GroEL/ES also increased the amount of active β-gal produced but had little positive effect on active mCherry synthesis (Figure S6a and S7a). In contrast, the DnaK/DnaJ/GrpE system did not show much positive effect on the synthesis of all of the three proteins (Figure 3b, Figure S6b and S7b). This finding is different from the previous result which saying the DnaK system enhanced the expression of functional single-chain antibody but the GroEL/ES system didn't [36]. The contradiction between our findings indicates both GroEL/ES and DnaK/DnaJ/GrpE contribute to protein folding and increase the level of functional protein produced but with their own specificity on different proteins.

In fact, the fidelity of protein synthesis is sensitive to changes in magnesium concentration and at a high Mg^2+^ concentration, ribosomes might become stuck at a translocation step [29]. In our case, an increase of only 4 mM Mg^2+^ in the commercialized PURE system reduced the yield of functional Fluc by 70% (Figure 4b). Using our optimized system dramatically altered the picture: when Mg^2+^ concentration was increased by 4 mM, the yield of functional Fluc was not reduced (Figure 4c).

Our finding that the crowding agent BSA can significantly enhance PURE system productivity is not surprising since transcriptions and translations *in vivo* occur in macromolecular crowding environments. Adding BSA enhanced the association of biomolecules due to its excluded volume effect, which increased the effective concentrations of the enzymes and biomolecular reactants [40], [41], and so altered the rates and equilibrium constants of their reactions [42]. In addition, BSA as a crowding agent, also increased the solution viscosity, thus could dramatically reduce the diffusion coefficients of biomolecules by factors up to 10 fold [43]. Our observation was in agreement with other studies on macromolecular crowding effects on biochemical reactions involving DNA and protein association. For example, DNA replication [44]–[46], ligation [47], PCR [48], restriction digestion [49], nuclease degradation [50] and transcription [30] all can be facilitated by crowding agents because crowding agents can dramatically increase the association between enzymes and DNA. Here we were able to move a step further by showing that coupled transcription and translation can be significantly boosted by BSA as a whole, whereas a previous study instead used a two-stage system in which macromolecular crowding was used to enhance a transcription reaction, after which the mRNA was purified and transferred to a second translation reaction [30]. We also found that adding PEG-6000 rather than BSA reduced rather than increased productivity, presumably by causing proteins to aggregate.

Mimicking the intracellular environment of living cells has produced significant improvements in PURE system expression; however, there are some new factors that remain to be addressed. A larger parameter space of the concentrations of other factors present in the PURE system, such as tRNA synthetases, can also be tested. The recently identified Elongation factor P can prevent ribosome from stalling during synthesis of proteins containing consecutive prolines, such as PPG, PPP, or longer proline strings, in natural and engineered model proteins [31], [51]. We observed that the PURE system produces half translated products (Figure S10) most likely due to ribosome stalling on mRNA. Therefore, besides EF-P, there might be other unidentified factors also helping rescue stalled ribosomes. And all of these factors can serve as supplements for PURE system translation. Finally, it is also worth pointing out that the efficiency of CFPS is also limited by energy supply and inhibitory by-products generated from translation process [52], [53]. This problem can possibly be resolved by a continuous-exchange PURE system.

In summary, we believe our improved PURE system is an attractive platform for *in vitro* protein synthesis. It produces more than 5 fold active Fluc compared with the original system, and our work makes the PURE system more cost-efficient with a comparable productivity and cost level to the *E. coli* crude extract CFPS. Our results will have profound implications for systems and synthetic biology by enabling better reproducibility of gene transcription and translation process in an *in vitro* setting. Finally, by reassembling the “central-dogma” pathway of molecular biology with purified components, our system can serve as a basis for construction of a minimal protein based self-replicating system [54].

## Materials and Methods

### Media, chemicals, and reagents

Unless specified, all chemicals were obtained from Sigma-Aldrich. PEG-6000 (molecular weight: 6 kDa) was purchased from Fisher (Pittsburg, PA). A 50% (w/v) stock solution of PEG-6000 was prepared in nuclease-free water before being used in experiments. BSA was purchased from New England Biolabs. GroEL/ES were obtained from Takara; DnaJ was obtained from Accurate Chemical & Scientific Corporation, DnaK and GrpE were obtained from Novus Biologicals. Tryptone and yeast extract were obtained from BD Difco. For protein purification, liquid cultures of all strains were grown in SB media (24 g/L tryptone, 12 g/L yeast extract, 5 g/L glucose, 2 g/L NaH_2_PO_4_, 16.4 g/L K_2_HPO_4_-3H_2_O, 4 mL/L glycerol). Protein purifications were carried out with ÄKTAprime (GE Healthcare) equipped with 5 mL HisTrap HP column (GE Healthcare). Purified protein concentrations were determined by standard Bradford assay (Bio-Rad).

### Molecular cloning

The Fluc gene was cloned into the NcoI and XhoI restriction sites of pIVEX-2.3d (5PRIME). MCherry and lacZ were cloned into the NdeI and XhoI restriction sites of pET-24b (Novagen). PIVEX 2.3d-Fluc, pET-24b mCherry and pET-24b lacZ were purified with phenol: chloroform: isoamyl alcohol extraction and ethanol precipitation before being used for *in vitro* transcription. EF-4 gene (lepA) sequence was amplified by PCR with primers 5′GGAATTCCATATGAAGAATATACGTAACTTTTCGAT3′ and 5′CCGCTCGAGTTAGTGATGGTGATGGTGATGTTTGTTGTCTTTGCCGACGTG3′ from *E. coli* MG1655 genomic DNA (ATCC) and the PCR products were inserted into the NdeI and XhoI restrictions site of pET-24b. Plasmids encoding IF1, 2, 3, EF-Tu, Ts, G, RF1, 2, 3 and RRF were provided by T. Ueda. The vectors pLG1 and pLG2 (with and without a stop codon, respectively) for expressing HaloTagged proteins were derived from pFN18K (Promega). Protein genes were inserted into a polylinker region between the sequences of an N-terminal HaloTag domain and a C-terminal 171-amino-acid alpha-helical spacer excised from *E. coli* TolA domain II. Specifically, the gene of mCherry was inserted by using by NdeI and SacI restriction sites and the genes of *E. coli* fusion proteins, EntE-mCherry and EntF-mCherry, were inserted by using XhoI and SacI restriction sites. Linear DNA templates encoding HaloTagged proteins were generated by PCR, purified and then used for PURE system reactions. PCR products started from T7 promoter and ended right before or at stop codon of the HaloTagged proteins.

### Over-expression and purification of IF1, 2, 3, EF-Tu, Ts, G, RF1, 2, 3, RRF and EF4

IF1, 2, 3, EF-Tu, Ts, G, RF1, 2, 3 and RRF were over-expressed and purified as described in [20] and [32]. PET-24b EF-4 were transformed to NEB BL21 (DE3) (New England Biolab) strain. Cells were grown in 300 mL SB with 50 µg/mL kanamycin at 37 °C, induced with 1 mM IPTG when OD_600_ reached 0.5, and further incubated at 37 °C for 4 hours before harvest. Cell paste was lysed by 1 mL BugBuster 10× Protein Extraction Reagent (EMD Chemical), with 100 µL Halt-protease inhibitor (Thermo-Fisher), 6 mM β-ME, and 20 mM imidazole-HOAc (pH 7.4). Cell debris was removed by centrifugation at 150,000× *g* for 25 min twice. The supernatant was loaded to a Ni-NTA column and washed with wash buffer (20 mM Tris-HOAc, pH 7.6, 30 mM NH_4_Cl, 150 mM KCl, and 150 mM NaCl) containing 20 mM Imiazole-HOAc (pH 7.4). The His-tagged EF-4 was eluted with a linear gradient from 20 mM to 400 mM Imidazole-HOAc in wash buffer, pooled, concentrated with Amicon-Ultra-4 concentrator with 3 K MWCO, and dialyzed against 1 L of stock buffer (20 mM Tris-HOAc, pH 7.6, 30 mM NH_4_Cl, 150 mM KCl, 15 mM Mg(OAc)_2_, 6 mM βME, and 10 µM GDP) for 3 hours twice. Purified EF-4 was added to 20% glycerol and stored at −80°C.

### PURE transcription and translation optimization

PURE system kits were purchased from New England Biolabs. 25 µL reactions were carried out following the PURE system manual with 10 µL solution A, 7.5 µL solution B, 0.8 U/µL Murine Rnase Inhibitor (New England Biolabs), 10 µg/mL pIVEX 2.3d-Fluc, or 5 µg/mL pET-24b mCherry or 3 µg/mL pET-24b lacZ and proper amount of supplement factors as indicated in the text. Reactions were incubated at 37 °C for 2 hours. Fluc activity was measured by Promega Luciferase Assay System kit. β-gal activity was measured by Galacto-Light Plus β-Galactosidase Reporter Gene Assay System (Life Technologies). Chemical luminescence in relative luminescent units (RLUs) and fluorescence in relative fluorescence units (RFU) were measured by a microplate reader (SpectraMax M5, Molecular Devices).

### mRNA quantification

After the reaction, the DNA template was removed by digestion with 0.5 µl DNase I (≥2,500 U/ml, Thermo scientific) at 37°C for 15 min. Total mRNA was then quantified with the Quant-iT RiboGreen RNA Reagent and Kit (Invitrogen, CA) according to the manufacturer's instructions.

### Labelling of in vitro translated proteins with a fluorescent HaloTag ligand

To further examine full-length and incomplete products of *in vitro* translation in PURE system, three N-terminal HaloTagged recombinant proteins of various sizes were *in vitro* translated and labelled with a fluorescent TMR reporter. To answer if the ribosome is recycled during the translation, two expression constructs with or without a stop codon were prepared for each protein. Linear DNA templates were used for PURE system reactions. Reactions were incubated at 37°C for 2 hours, and translated proteins were incubated with 5 µM Halo-TMR (Promega) in a 1× PBS buffer at room temperature for 30 min prior to SDS-PAGE analysis. The gel was scanned at 580 nm (excitation at 532 nm) by Typhoon Trio Imager (GE Healthcare) and a fluorescence gel image was analyzed with ImageQuant TL software.

## Supporting Information

Figure S1 Assessment of purified C-terminal His-tagged EF4. BenchMark Protein Ladder (Life Technologies), *E. coli* cell lysate overexpressing C-terminal His-tagged EF4, flowthrough and eluted fractions of EF4 after Ni-NTA purification were analyzed on 4-12% Bis-Tris PAGE gel, stained by Coomassie-blue. EF4 with a MW of 66.57 kD migrated as expected.
(TIF)

Figure S2 Optimization of PURE system as measured by active mCherry produced by supplementing different concentrations of EF-Tu, Ts, G; EF4; RF1, 2, 3 and RRF. (**a**). Active mCherry produced at different EF-Ts, Tu and G concentrations. The table below shows the actual concentration increase of EF-Ts, Tu and G in the PURE system. (**b**). Active mCherry produced at different EF4 concentrations. (**c**). Active mCherry produced at different RF1, 2, 3 and RRF concentrations. The table below shows the actual concentration increase of RF1, 2, 3 and RRF in the PURE system. MCherry activities were measured by relative fluorescence unit and PURE system reaction without supplement was set as control. Error bars are ± standard deviations, with n = 3.
(TIF)

Figure S3 Optimization of PURE system as measured by active β-gal produced by supplementing different concentrations of EF-Tu, Ts, G; EF4; RF1, 2, 3 and RRF. (**a**). Active β-gal produced at different EF-Ts, Tu and G concentrations. The table below shows the actual concentration increase of EF-Ts, Tu and G in the PURE system. (**b**). Active β-gal produced at different EF4 concentrations. (**c**). Active β-gal produced at different RF1, 2, 3 and RRF concentrations. The table below shows the actual concentration increase of RF1, 2, 3 and RRF in the PURE system. β-gal activities were measured in relative luminescence unit by Galacto-Light Plus β-Galactosidase Reporter Gene Assay System (Life Technologies) and PURE system reaction without supplement was set as control. Error bars are ± standard deviations, with n = 3.
(TIF)

Figure S4 MCherry and β-gal synthesis in the PURE system with BSA as a macromolecular crowding agent. (**a**) Active mCherry produced at different concentrations of BSA. MCherry activities were measured in relative fluorescence unit. (**b**) Active β-gal produced at different concentrations of BSA. β-gal activities were measured in relative luminescence unit by Galacto-Light Plus β-Galactosidase Reporter Gene Assay System (Life Technologies). PURE system reaction without supplement was set as control. Error bars are ± standard deviations, with n = 3.
(TIF)

Figure S5 Assessment of Fluc yield at different concentrations of GroEL/ES in PURE system. PIVEX 2.3d-Fluc was added to PURE system reaction mixture with different concentrations (from control 0 µM to 6 µM) of GroEL/ES. After 2 hours incubation at 37°C, each reaction was analyzed directly on 4–12% Bis-Tris PAGE gel, stained by Coomassie-blue. The expected migration bands of GroEL/ES and firefly luciferase are marked on the gel.
(TIF)

Figure S6 MCherry synthesis in the PURE system with chaperone systems GroEL/ES and DnaK/DnaJ/GrpE. (**a**). Active mCherry produced at different GroEL/GroES concentrations. (**b**). Active mCherry produced at different DnaK/DnaJ/GrpE concentrations. MCherry activities were measured in relative fluorescence unit and PURE system reaction without supplement was set as control. Error bars are ± standard deviations, with n = 3.
(TIF)

Figure S7 β-gal synthesis in the PURE system with chaperone systems GroEL/ES and DnaK/DnaJ/GrpE. (**a**). Active β-gal produced at different GroEL/GroES concentrations. (**b**). Active β-gal produced at different DnaK/DnaJ/GrpE concentrations. β-gal activities were measured in relative luminescence unit by Galacto-Light Plus β-Galactosidase Reporter Gene Assay System (Life Technologies) and PURE system reaction without supplement was set as control. Error bars are ± standard deviations, with n = 3.
(TIF)

Figure S8 MCherry and β-gal synthesis in the PURE system with their best combination of enzyme factor concentrations and two different tRNA concentrations. (**a**) Active mCherry produced with the best combination of EF-Tu, Ts, G; EF4; RRF, RF1, RF2, RF3 and BSA concentrations and two different tRNA concentrations. MCherry activities were measured in relative fluorescence units. (b) Active β-gal produced with the best combination of EF-Tu, Ts, G; EF4; RRF, RF1, RF2, RF3; GroEL/ES and BSA concentrations and two different tRNA concentrations. β-gal activities were measured in relative luminescence unit by Galacto-Light Plus β-Galactosidase Reporter Gene Assay System (Life Technologies). Error bars are ± standard deviations, with n = 3.
(TIF)

Figure S9 Optimization of PURE system as measured by functional Fluc produced by addingdifferent concentrations of RRF, EF-G, IF1, 2 and 3. (**a**). Active Fluc produced at different RRF, EF-G and IF3 concentrations. The table below shows the actual concentration increase of RRF, EF-G and IF3 in PURE system. (**b**). Active Fluc produced at different IF1, 2 and 3 concentrations. The table below shows the actual concentration increase of IF1, 2 and 3 in PURE system. Fluc activities were measured in relative luminescence unit by luciferase assay and PURE system reaction without supplement was set as control. Error bars are ± standard deviations, with n = 3.
(TIF)

Figure S10 Assessment of PURE system translation by production of HaloTag fusion proteins using linear DNA templates. Left panel shows the three HaloTag fusion proteins with and without stop codon (90 kD, 160 kD, 220 kD) expressed in PURE system on SDS-PAGE gel, stained by Coomassie-blue. The right panel shows the same gel but with samples incubated with HaloTag TMR Ligand. The gel was scanned by a typhoon scanner with filter set (555 nmEx/580 nmEm). Therefore half-translated products can be shown via scanning on the gel. Lane 1 is protein marker. Lane 2, 4, 6 are HaloTag fusion proteins without stop codon. Lane 3, 5, 7 are HaloTag fusion proteins with stop codon. Lane 8 is a negative control with no DNA template. (HT: HaloTag; Ch: mCherry. TolA is a C-terminal 171-amino-acid alpha-helical spacer excised from *E. coli* TolA domain II. EntE and EntF are multidomain enzymes from *E. coli* enterobactin biosynthetic pathway.)
(TIF)
