# Supplementary figures and images for: Improved Cell-Free RNA and Protein Synthesis System
Source: PLoS One. 2014 Sep 2;9(9):e106232. doi: 10.1371/journal.pone.0106232 (PMC4152126; doi:10.1371/journal.pone.0106232)

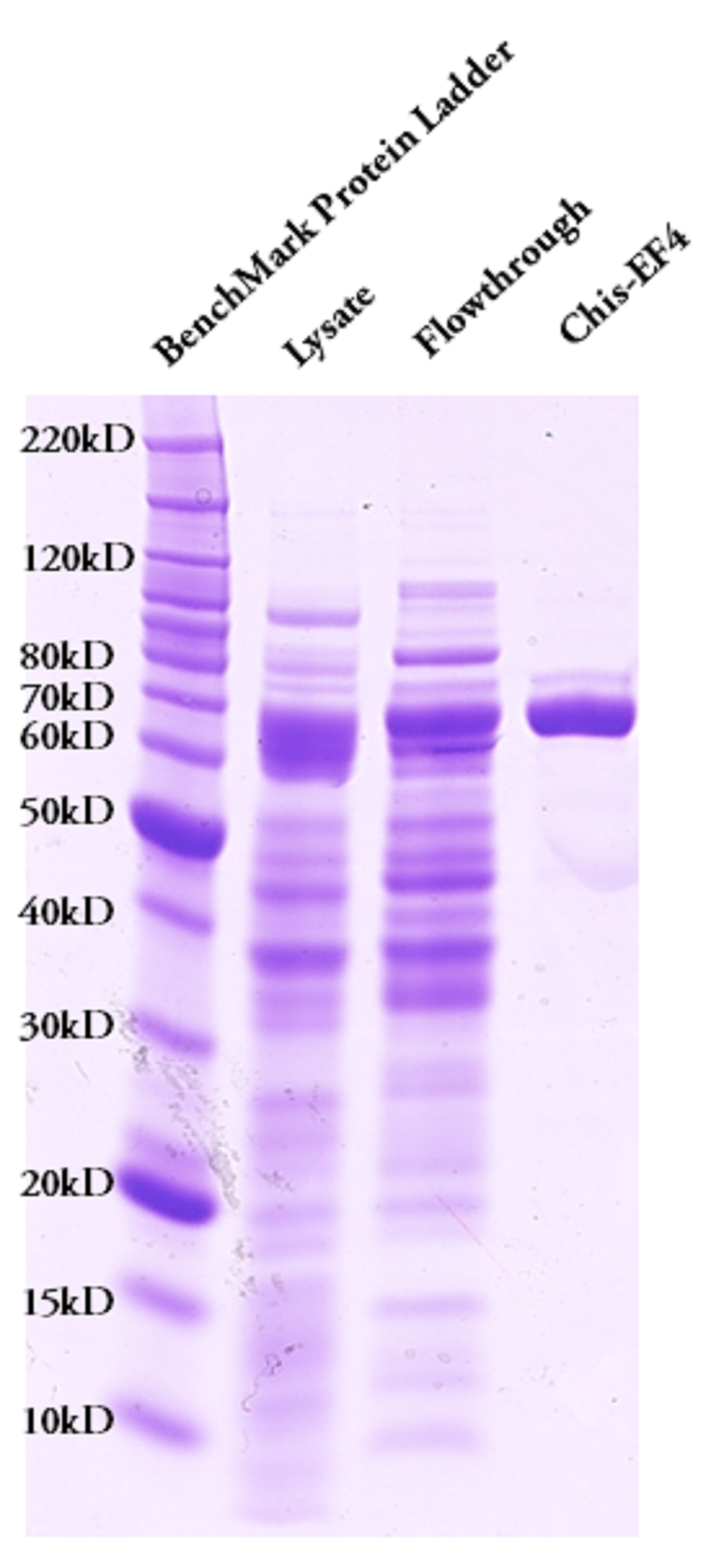

Supplement: Figure S1 — Assessment of purified C-terminal His-tagged EF4. BenchMark Protein Ladder (Life Technologies), E. coli cell lysate overexpressing C-terminal His-tagged EF4, flowthrough and eluted fractions of EF4 after Ni-NTA purification were analyzed on 4-12% Bis-Tris PAGE gel, stained by Coomassie-blue. EF4 with a MW of 66.57 kD migrated as expected. (TIF) [file pone.0106232.s001.tif]

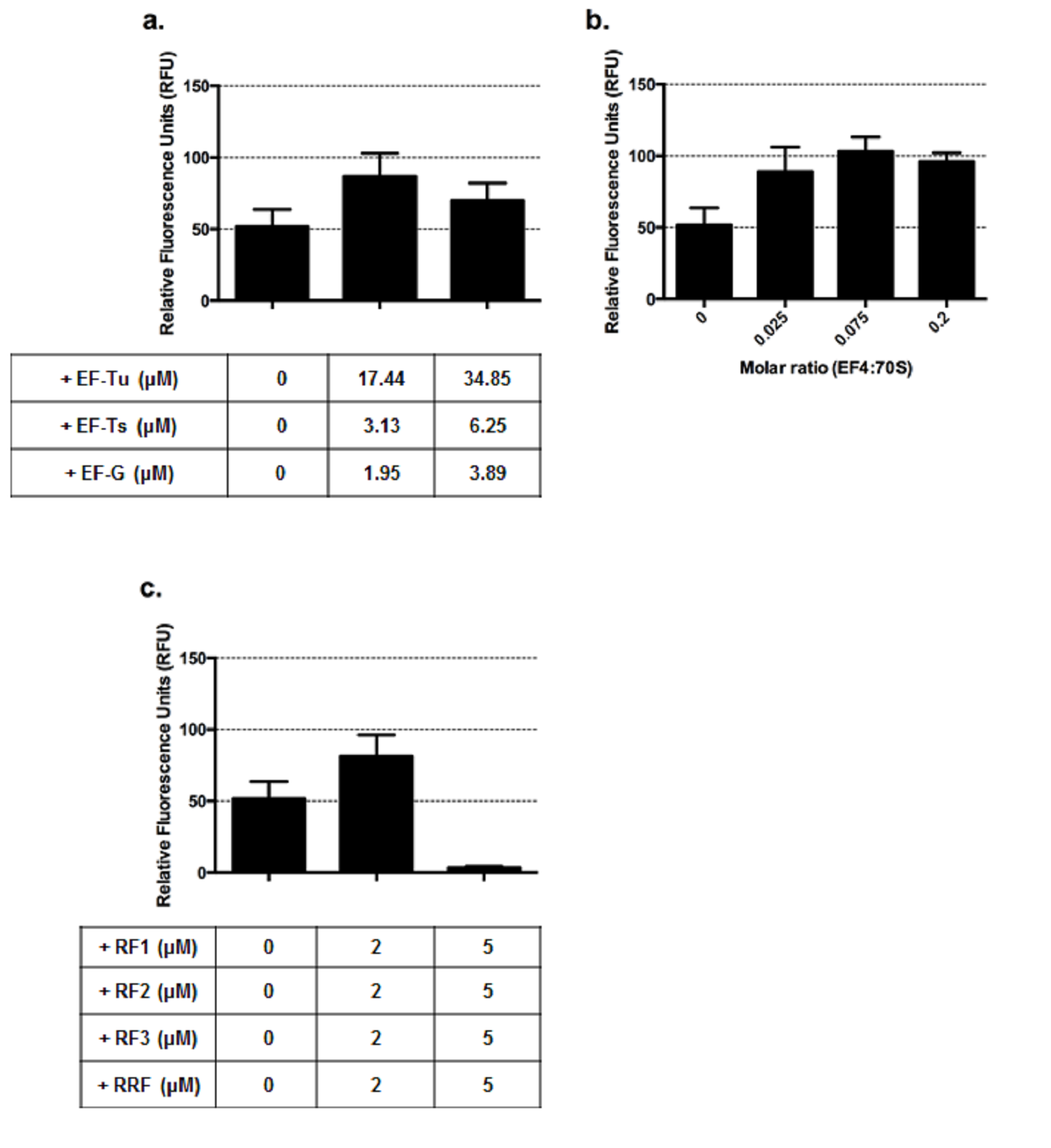

Supplement: Figure S2 — Optimization of PURE system as measured by active mCherry produced by supplementing different concentrations of EF-Tu, Ts, G; EF4; RF1, 2, 3 and RRF. (a). Active mCherry produced at different EF-Ts, Tu and G concentrations. The table below shows the actual concentration increase of EF-Ts, Tu and G in the PURE system. (b). Active mCherry produced at different EF4 concentrations. (c). Active mCherry produced at different RF1, 2, 3 and RRF concentrations. The table below shows the actual concentration increase of RF1, 2, 3 and RRF in the PURE system. MCherry activities were measured by relative fluorescence unit and PURE system reaction without supplement was set as control. Error bars are ± standard deviations, with n = 3. (TIF) [file pone.0106232.s002.tif]

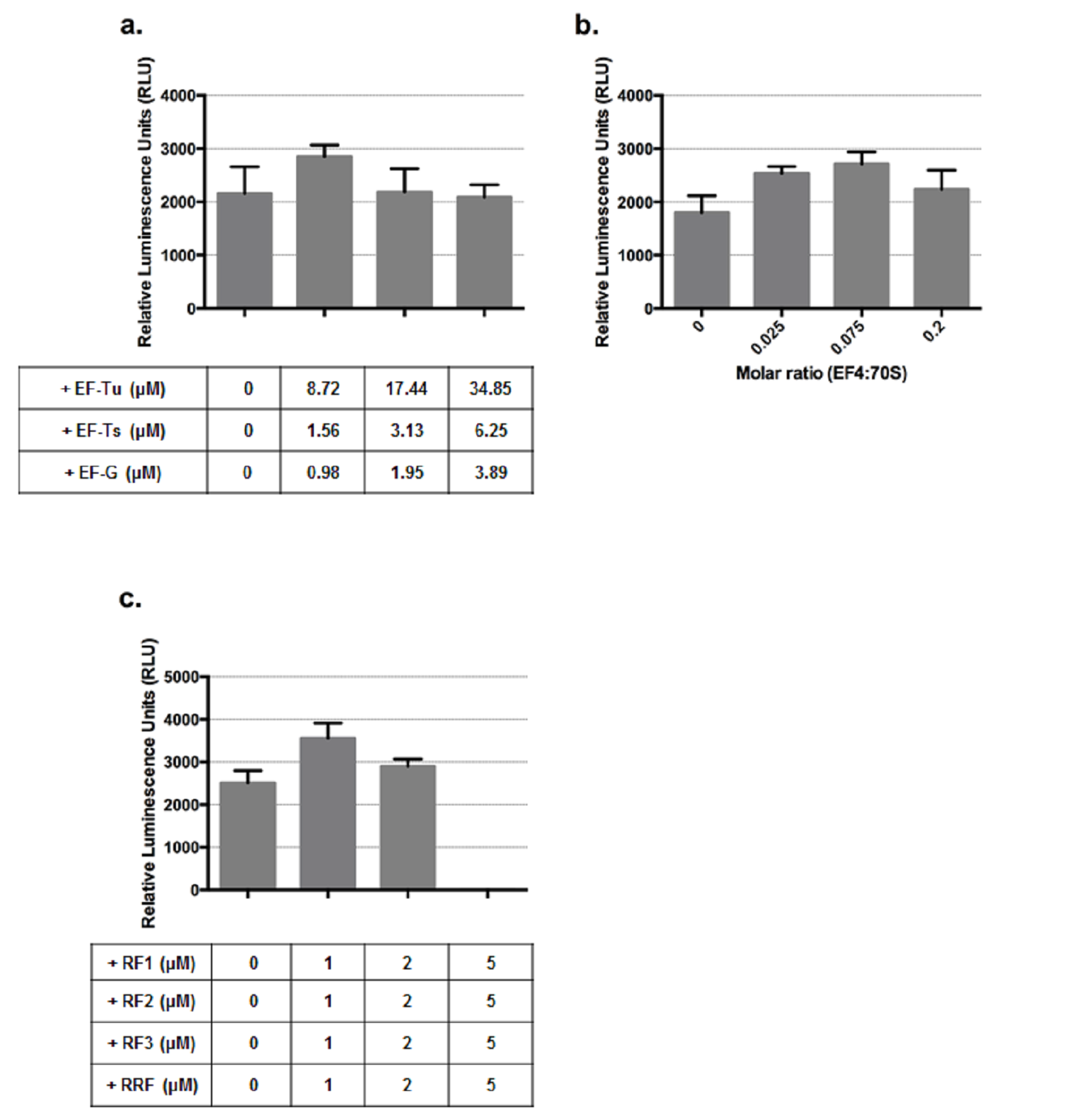

Supplement: Figure S3 — Optimization of PURE system as measured by active β-gal produced by supplementing different concentrations of EF-Tu, Ts, G; EF4; RF1, 2, 3 and RRF. (a). Active β-gal produced at different EF-Ts, Tu and G concentrations. The table below shows the actual concentration increase of EF-Ts, Tu and G in the PURE system. (b). Active β-gal produced at different EF4 concentrations. (c). Active β-gal produced at different RF1, 2, 3 and RRF concentrations. The table below shows the actual concentration increase of RF1, 2, 3 and RRF in the PURE system. β-gal activities were measured in relative luminescence unit by Galacto-Light Plus β-Galactosidase Reporter Gene Assay System (Life Technologies) and PURE system reaction without supplement was set as control. Error bars are ± standard deviations, with n = 3. (TIF) [file pone.0106232.s003.tif]

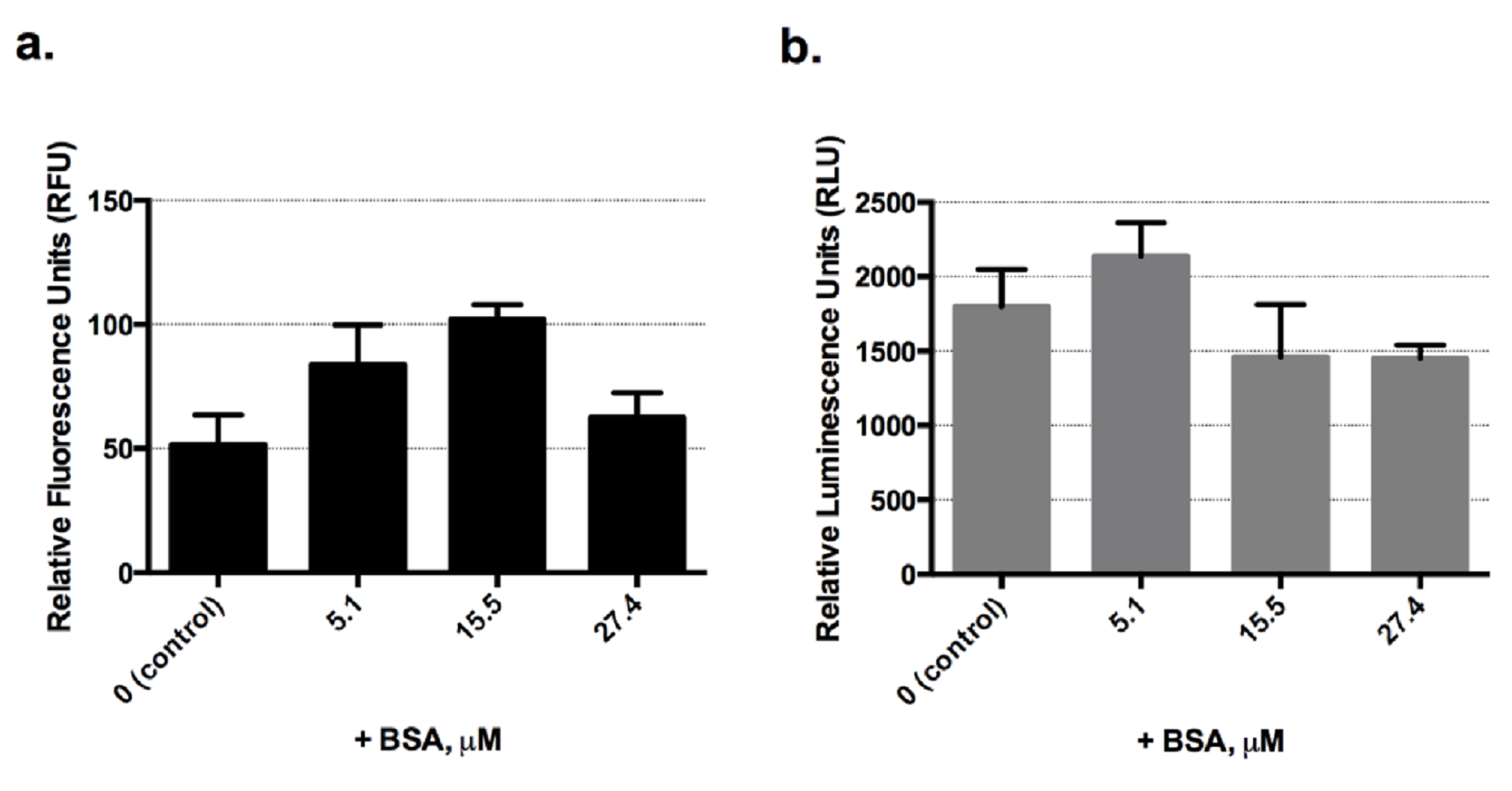

Supplement: Figure S4 — MCherry and β-gal synthesis in the PURE system with BSA as a macromolecular crowding agent. (a) Active mCherry produced at different concentrations of BSA. MCherry activities were measured in relative fluorescence unit. (b) Active β-gal produced at different concentrations of BSA. β-gal activities were measured in relative luminescence unit by Galacto-Light Plus β-Galactosidase Reporter Gene Assay System (Life Technologies). PURE system reaction without supplement was set as control. Error bars are ± standard deviations, with n = 3. (TIF) [file pone.0106232.s004.tif]

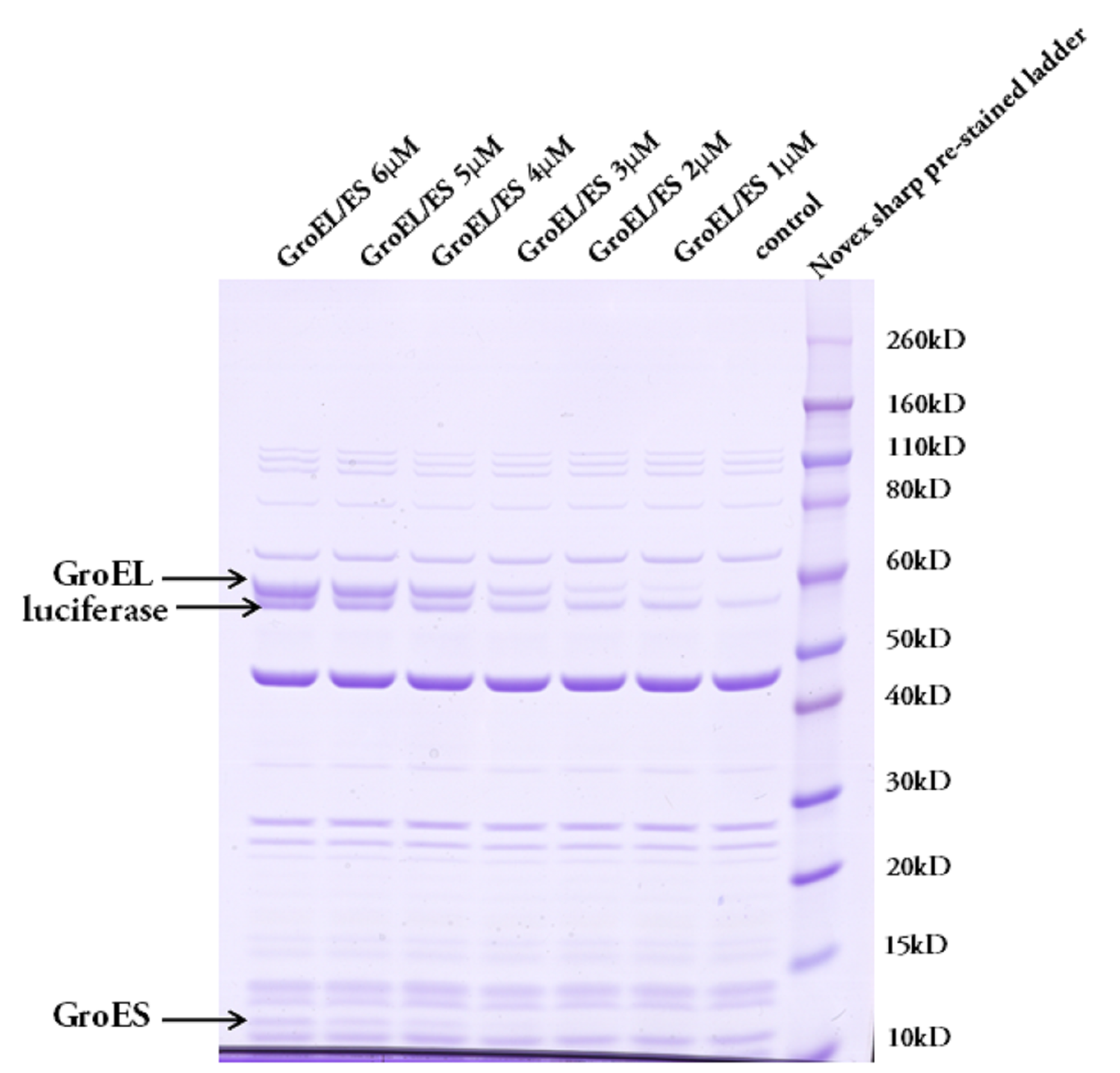

Supplement: Figure S5 — Assessment of Fluc yield at different concentrations of GroEL/ES in PURE system. PIVEX 2.3d-Fluc was added to PURE system reaction mixture with different concentrations (from control 0 µM to 6 µM) of GroEL/ES. After 2 hours incubation at 37°C, each reaction was analyzed directly on 4–12% Bis-Tris PAGE gel, stained by Coomassie-blue. The expected migration bands of GroEL/ES and firefly luciferase are marked on the gel. (TIF) [file pone.0106232.s005.tif]

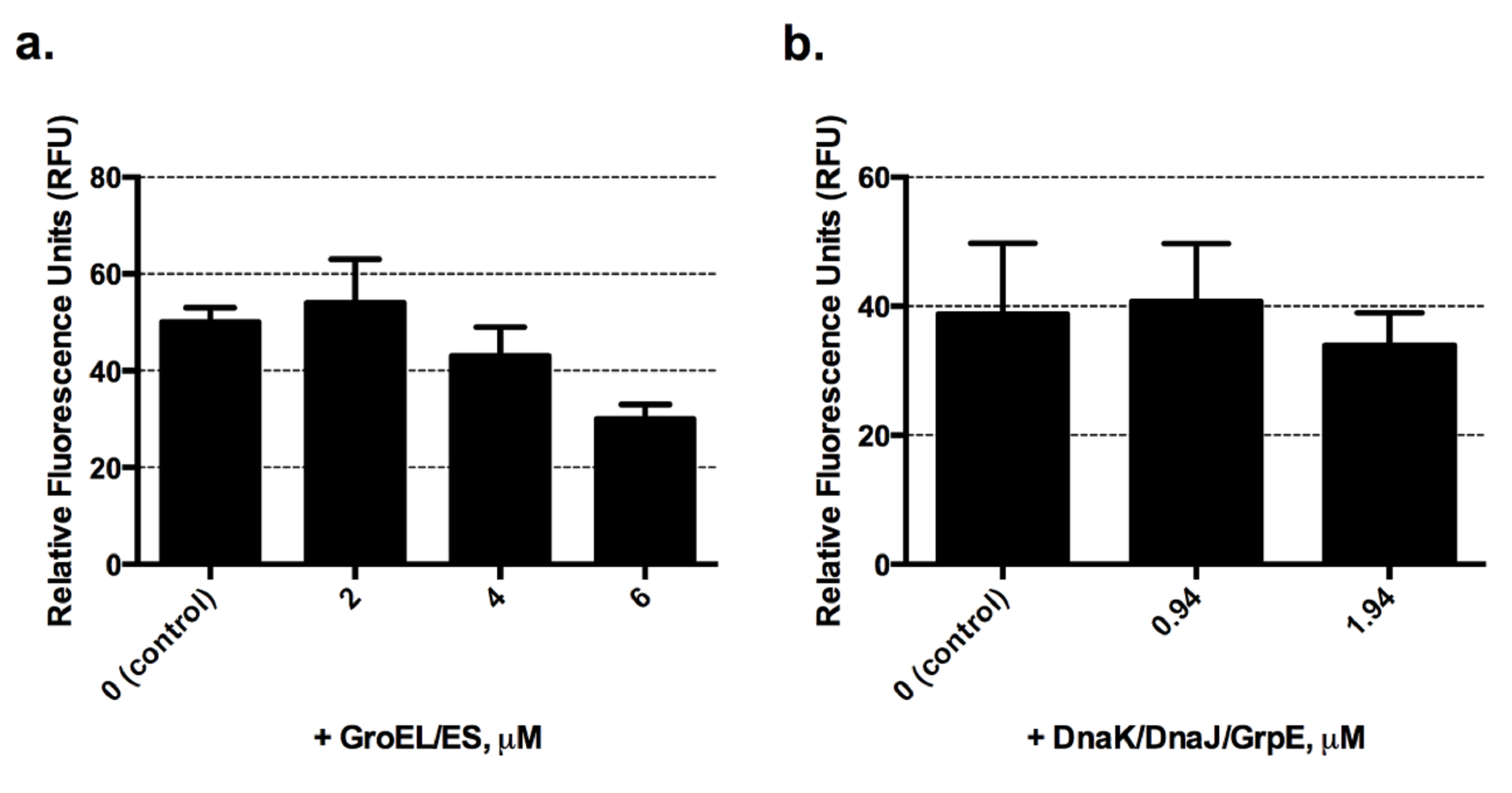

Supplement: Figure S6 — MCherry synthesis in the PURE system with chaperone systems GroEL/ES and DnaK/DnaJ/GrpE. (a). Active mCherry produced at different GroEL/GroES concentrations. (b). Active mCherry produced at different DnaK/DnaJ/GrpE concentrations. MCherry activities were measured in relative fluorescence unit and PURE system reaction without supplement was set as control. Error bars are ± standard deviations, with n = 3. (TIF) [file pone.0106232.s006.tif]

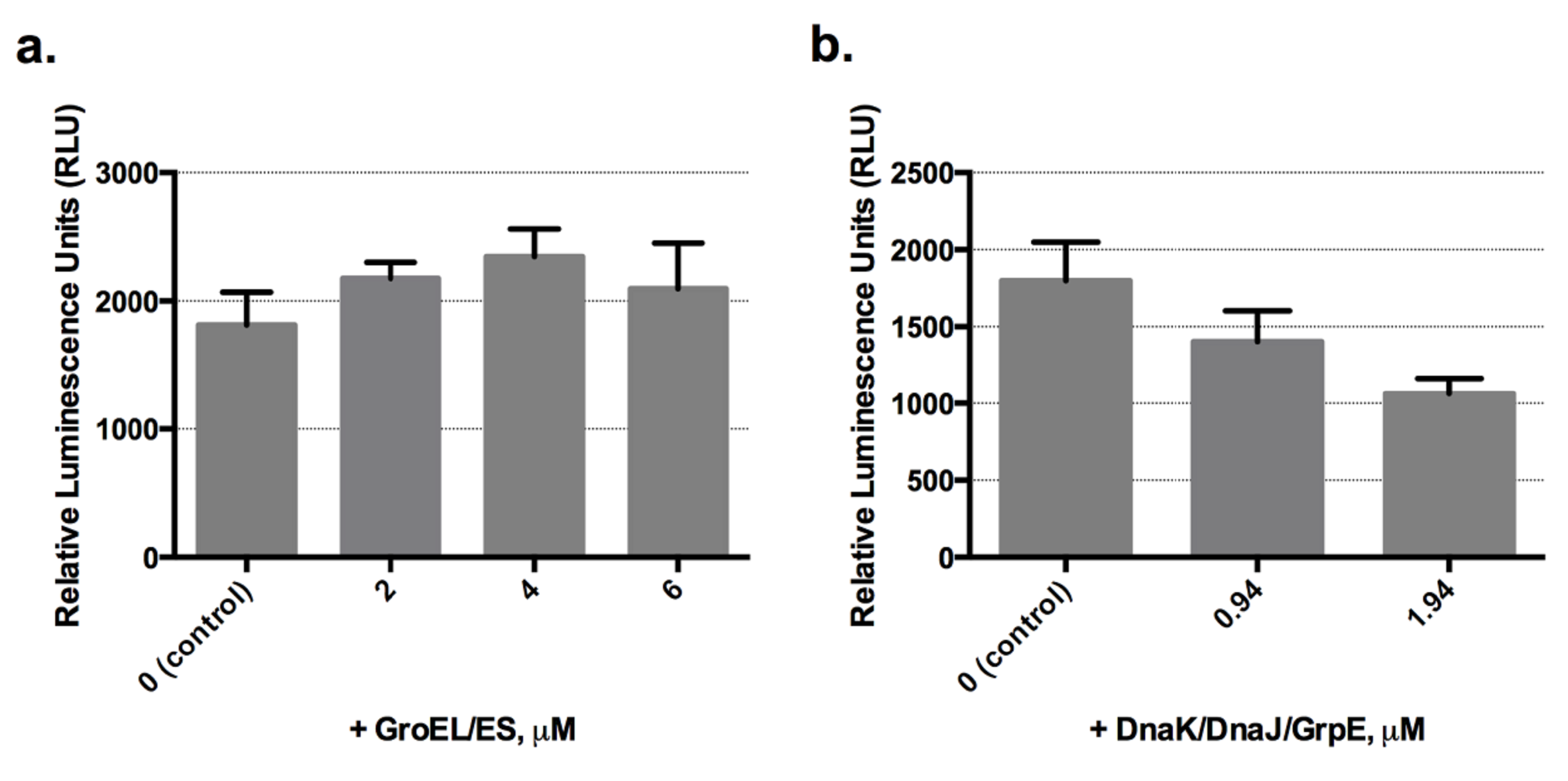

Supplement: Figure S7 — β-gal synthesis in the PURE system with chaperone systems GroEL/ES and DnaK/DnaJ/GrpE. (a). Active β-gal produced at different GroEL/GroES concentrations. (b). Active β-gal produced at different DnaK/DnaJ/GrpE concentrations. β-gal activities were measured in relative luminescence unit by Galacto-Light Plus β-Galactosidase Reporter Gene Assay System (Life Technologies) and PURE system reaction without supplement was set as control. Error bars are ± standard deviations, with n = 3. (TIF) [file pone.0106232.s007.tif]

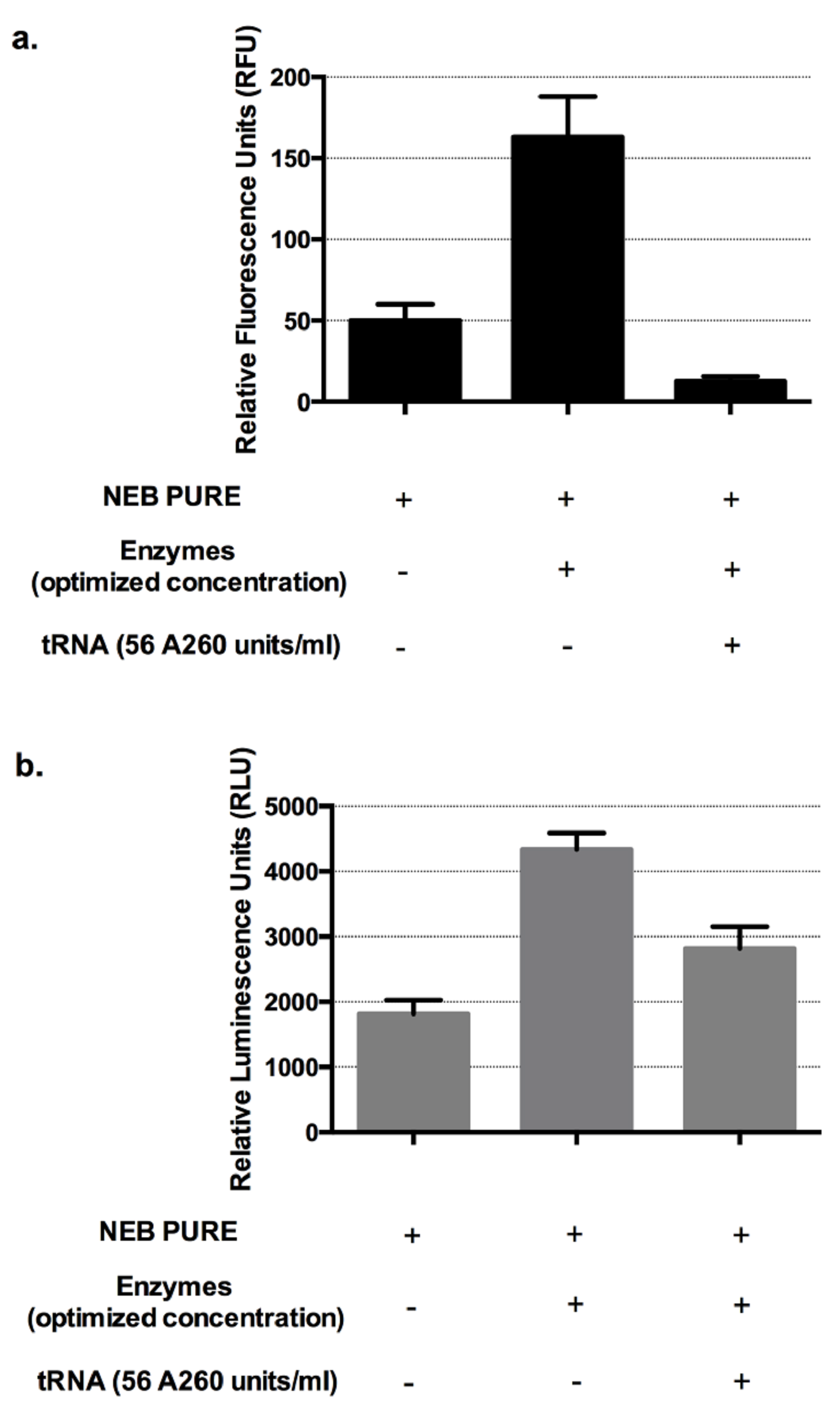

Supplement: Figure S8 — MCherry and β-gal synthesis in the PURE system with their best combination of enzyme factor concentrations and two different tRNA concentrations. (a) Active mCherry produced with the best combination of EF-Tu, Ts, G; EF4; RRF, RF1, RF2, RF3 and BSA concentrations and two different tRNA concentrations. MCherry activities were measured in relative fluorescence units. (b) Active β-gal produced with the best combination of EF-Tu, Ts, G; EF4; RRF, RF1, RF2, RF3; GroEL/ES and BSA concentrations and two different tRNA concentrations. β-gal activities were measured in relative luminescence unit by Galacto-Light Plus β-Galactosidase Reporter Gene Assay System (Life Technologies). Error bars are ± standard deviations, with n = 3. (TIF) [file pone.0106232.s008.tif]

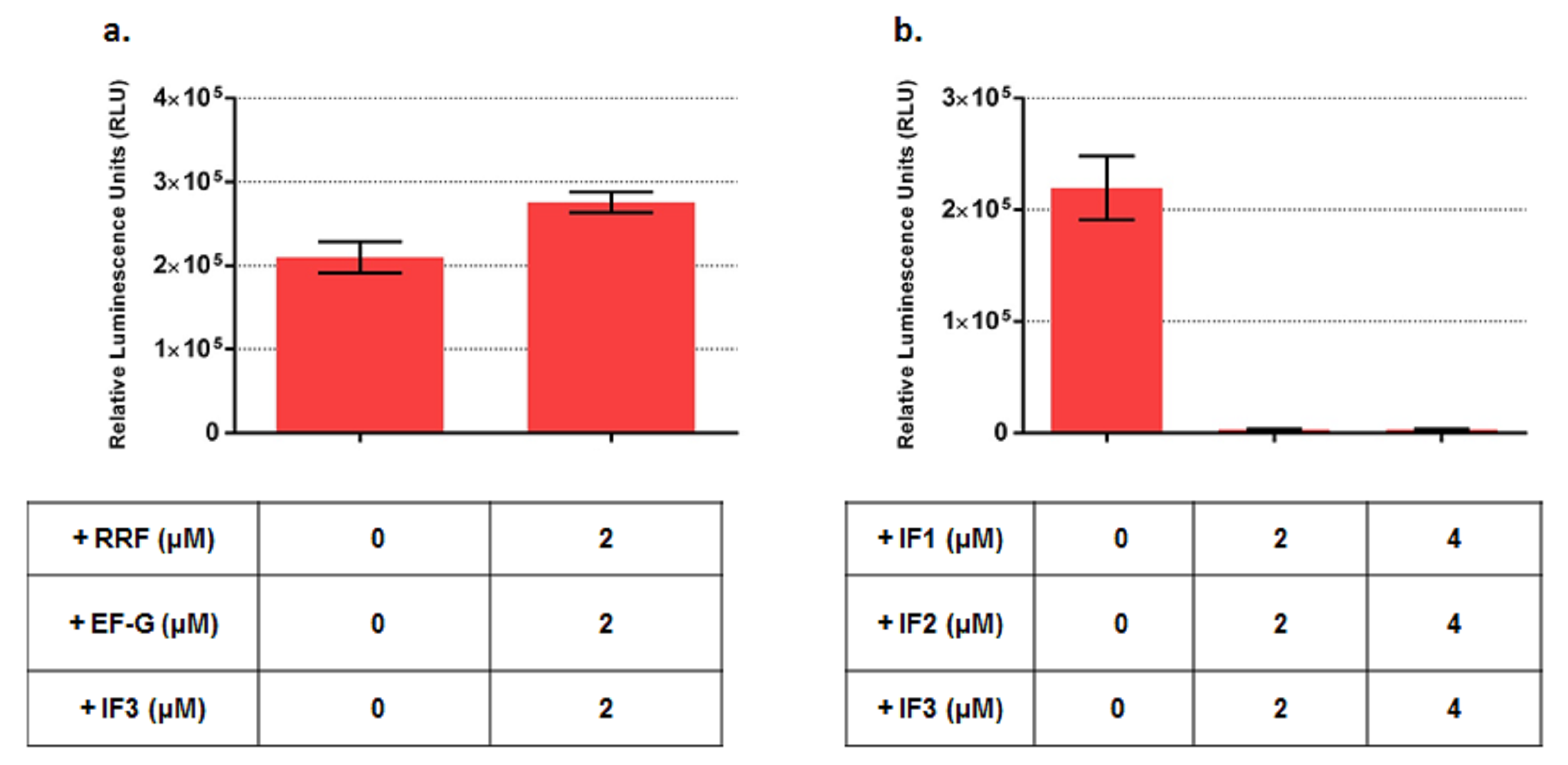

Supplement: Figure S9 — Optimization of PURE system as measured by functional Fluc produced by addingdifferent concentrations of RRF, EF-G, IF1, 2 and 3. (a). Active Fluc produced at different RRF, EF-G and IF3 concentrations. The table below shows the actual concentration increase of RRF, EF-G and IF3 in PURE system. (b). Active Fluc produced at different IF1, 2 and 3 concentrations. The table below shows the actual concentration increase of IF1, 2 and 3 in PURE system. Fluc activities were measured in relative luminescence unit by luciferase assay and PURE system reaction without supplement was set as control. Error bars are ± standard deviations, with n = 3. (TIF) [file pone.0106232.s009.tif]

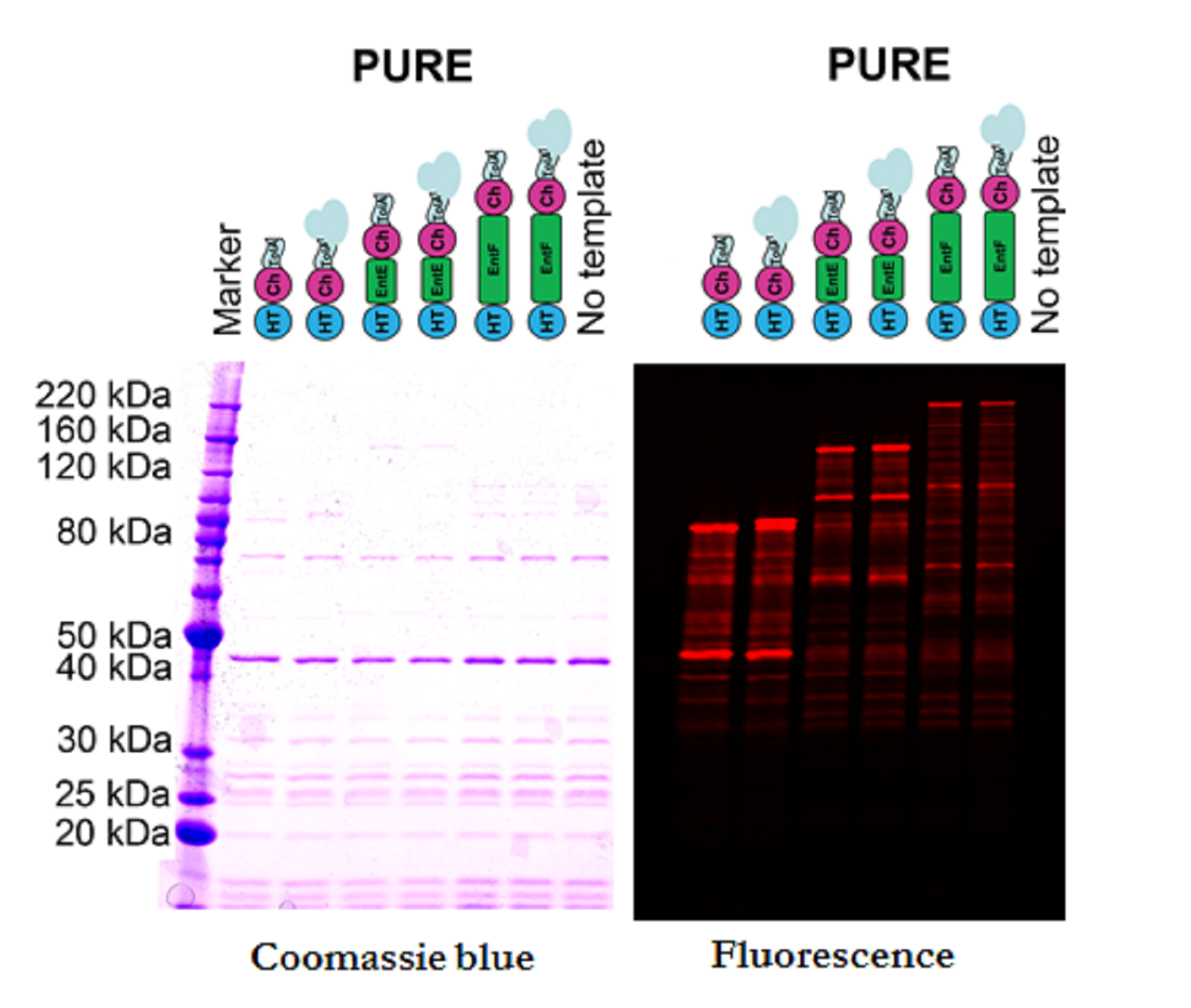

Supplement: Figure S10 — Assessment of PURE system translation by production of HaloTag fusion proteins using linear DNA templates. Left panel shows the three HaloTag fusion proteins with and without stop codon (90 kD, 160 kD, 220 kD) expressed in PURE system on SDS-PAGE gel, stained by Coomassie-blue. The right panel shows the same gel but with samples incubated with HaloTag TMR Ligand. The gel was scanned by a typhoon scanner with filter set (555 nmEx/580 nmEm). Therefore half-translated products can be shown via scanning on the gel. Lane 1 is protein marker. Lane 2, 4, 6 are HaloTag fusion proteins without stop codon. Lane 3, 5, 7 are HaloTag fusion proteins with stop codon. Lane 8 is a negative control with no DNA template. (HT: HaloTag; Ch: mCherry. TolA is a C-terminal 171-amino-acid alpha-helical spacer excised from E. coli TolA domain II. EntE and EntF are multidomain enzymes from E. coli enterobactin biosynthetic pathway.) (TIF) [file pone.0106232.s010.tif]
